# Psychometric properties of the German version of the Youth Psychopathic traits Inventory – short version

**DOI:** 10.1186/s12888-020-02943-z

**Published:** 2020-11-23

**Authors:** Cyril Boonmann, Tania Pérez, Marc Schmid, Jörg M. Fegert, Emanuel Jauk, Klaus Schmeck

**Affiliations:** 1 Child and Adolescent Psychiatric Research Department, Psychiatric University Hospitals, University of Basel, Wilhelm Klein-Strasse 27, 4002 Basel, Switzerland; 2 Department of Forensic Child and Adolescent Psychiatry, Psychiatric University Hospitals, University of Basel, Basel, Switzerland; 3 Child and Adolescent Psychiatric University Hospital, University of Ulm, Ulm, Germany; 4 Clinical Psychology and Behavioral Neuroscience, Dresden University of Technology, Dresden, Germany

**Keywords:** Psychopathic traits, CU-traits, YPI-S, Gender

## Abstract

**Background:**

The aim of the current study is to examine the psychometric properties of the German Version of the Youth Psychopathic traits Inventory-Short Version (YPI-S).

**Methods:**

A sample of 856 adolescents (age: 15–19) from the German-speaking part of Switzerland was included. All participants completed the 50-item YPI, of which we derived the 18 items of the YPI-S. Furthermore, participants completed the Massachusetts Youth Screening Instrument-Version-2 (MAYSI-2), as well a self-report delinquency questionnaire.

**Results:**

We were able to replicate a three-factor structure and found moderate to good internal consistency for the total score as well as for the three dimensions of the YPI-S. Measurement invariance across gender was established. Furthermore, we found positive small to medium correlations with both internalizing and externalizing mental health problems, substance abuse problems, and offending behavior.

**Conclusions:**

Our results suggest that the German version of the YPI-S is a reliable and valid screening instrument for psychopathic traits in both boys and girls from the general population in the German-speaking part of Switzerland.

## Background

In the last two decades, researchers have become increasingly interested in psychopathy or psychopathic traits in juveniles. As a result, for example, limited pro social emotions (a term related to callous-unemotional traits, one of the features of psychopathy) have been included as a specifier of Conduct Disorder (CD) in the 5th edition of the Diagnostic and Statistical Manual of Mental Disorders (DSM-5) [2] in order to capture a high-risk subgroup of juveniles with CD [19, 29].

The concept of psychopathy in juveniles is often described using a three [21] or four-factor model [26]. Although both models characterize psychopathy as a superficially charming, grandiose, and manipulative interpersonal style, a callous and unemotional affective experience, and impulsive, irresponsible behavior, criminogenic behavior is interpreted differently. Forth et al. [26] include it as the fourth factor, whereas Cooke and Michie [21] argue that criminogenic behavior is rather a consequence and not a core feature of psychopathy.

With the increased interest in this topic, the development of assessment tools for juveniles has also increased. A nowadays widely used instrument for this target group is the Youth Psychopathic traits Inventory (YPI) introduced by Andershed, Kerr, Stattin, and Levander [5]. This 50-item self-report questionnaire is designed to assess psychopathic traits (i.e., interpersonal, affective and behavioral traits) in non-referred youths. It consists of a total score, three underlying dimensions (Grandiose-Manipulative [GM], Callous-Unemotional [CU], and Impulsive-Irresponsible [II]), and 10 subscales (Dishonest Charm, Grandiosity, Lying, Manipulation, Callousness, Unemotionality, Remorselessness, Impulsiveness, Thrill-Seeking, and Irresponsibility).

However, as the instrument was considered to be time consuming, Van Baardewijk et al. [59] developed a shorter, 18-item, version: the YPI – Short Version (YPI-S). This short version has recently been translated into German, [60] and still needs to be validated in the German-speaking language area. The aim of the current paper is, therefore, to examine the psychometric properties of the YPI-S in the German-speaking part of Switzerland.

### Development and psychometric properties of the YPI

There is a considerable amount of work on the psychometric properties of the 50-item version of the YPI, beginning in 2002. In their initial publication, Andershed, Kerr, et al. [3, 5] found good reliability for the YPI total score, acceptable (CU and II) to good (GM) reliability for the underlying dimensions, and questionable (Callousness, Unemotionality, Remorselessness) to good (Dishonest charm, Lying, Manipulation) reliability for the subscales. Boys scored significantly higher on the YPI total score, all three dimensions, and most subscales than girls. Factor analyses showed that the three-factor model had a good fit to the data. In addition, the YPI total score and the three dimensions were found to correlate with various forms of conduct problems. The YPI total score was also found to be related to externalizing mental health problems (MHP). Furthermore, a psychopathic-like group (high on all three dimensions) generally had more externalizing MHP compared to other groups with low or average YPI dimension scores in both genders. Finally, within a group of adolescents with conduct problems, Andershed, Kerr, et al. [3, 5] were able to find a subgroup with higher levels of psychopathic traits in both genders. This subgroup scored significantly higher on externalizing MHP than juveniles with lower levels of psychopathic traits [3, 5].

Since then, the YPI has been implemented and validated in a wide range of mainly first world countries, including Germany [40] and Switzerland [51, 57], in non-referred [8, 15, 35, 38, 44, 51, 57], clinical [4, 23–25], and offending samples [9, 16, 20, 40, 48, 52, 56].

Previous studies generally confirmed the proposed three-factor structure in general, as well as in boys and in girls [16, 22, 35, 48, 51, 52, 56, 57]. The internal consistency of the total score and the underlying dimensions was generally acceptable to excellent [4, 8, 15, 16, 20, 22, 35, 40, 44, 48, 51, 52, 56, 57], although some studies found lower consistencies for the CU [35, 52] and the II dimension [4, 8].

The instrument generally showed moderate to strong significant associations with other measures of psychopathic traits/psychopathy, such as the Antisocial Process Screening Device [APS D[28];] [16, 48, 52], and to a lesser extent the Psychopathy Checklist: Youth Version [PCL:YV [26];] [4, 9, 23, 56]. Furthermore, the YPI total score and the dimension scores were weakly to strongly related to various types of offending behavior [16, 22, 24, 44, 48, 52, 57], and weakly to moderately to externalizing MHP and substance use problems [16, 44, 48, 52], although CU traits were not always related to substance use problems [16, 35]. Results with regard to internalizing MPH were mixed, with on the one hand, a generally weak to moderate positive relationship [16, 44, 52] and on the other hand, a negative weak to moderate association [48, 56].

Finally, the predictive validity of the YPI for future antisocial/delinquent behavior showed mixed results, not only between studies but also within studies [9, 15, 18, 23, 56]. Results, however, were difficult to compare, mainly due to a large variation in information sampling, such as length of follow-up time, information source (self-report vs. official records), or outcome variable (e.g., institutional infractions, recidivism, relational violence). In accordance with common criminological knowledge, it seems likely that there is a relationship between YPI measured psychopathic traits and future antisocial/delinquent behavior, but this relationship disappears when controlled for other known predictors of recidivism, such as previous offenses and age at first offense [15].

### Development and psychometric properties of the YPI-S

Although the YPI shows a number of good properties (developed to avoid social desirability, three-factor structure in line with theoretical models, good to excellent internal consistency, seems to work well in both boys and girls, valid in community samples as well as in forensic and other institutional setting samples, applicable to a wide age range), the instrument is considered to be time consuming [59]. Therefore, Van Baardewijk et al. [59] developed a psychometrically sound short version of this instrument, the YPI-S. Through stepwise selection using principal components analysis and content related arguments they reduced the number of items of the original 50-item YPI to 18 items, with the same three factor structure as the original instrument (in both boys and girls). The YPI-S (total score and its dimensions) showed satisfactory reliability, high significant correlation with the original YPI and similar correlations to conduct problems [59].

In recent years, in addition to Sweden, the YPI-S has been validated in Belgium [16, 18], China [62], Ghana [1], Italy [27], Portugal [48, 50], Spain [47], the Netherlands [58] and the United States [30]. In line with the YPI, a similar three-factor structure was found for the YPI-S (although there are also some indications for a bi-factor model, including a fourth general psychopathic traits factor [i.e., the total score], [62], a point of discussion that is also occasionally raised with the YPI [41, 62–64]). Some studies, however, found a low loading for the item: “*I have probably skipped school or work more than other people*.” on the behavioral dimension. Although the affective and behavioral dimension sometimes demonstrated marginally acceptable Cronbach’s alphas, the overall internal consistency was generally acceptable to good.

In addition, the number of studies investigating measurement invariance (MI) of the YPI-S across gender is still limited to date, although MI should be established to in order to meaningfully compare the observed test results between different groups [61]. The three-factor model of the YPI-S was found to show MI [17, 47] or partial MI [49] in general population youths. MI was also given for the bi-factorial model of the YPI-S [62].

The YPI-S usually showed moderate to strong positive relations with other psychopathic traits self-reports (e.g., APSD, Inventory of Callous-Unemotional Traits [ICU]), and a small to moderate relationship with especially externalizing MHP, substance use problems, and delinquent behavior. Results regarding the relationship with internalizing MHP were mixed, with some studies reporting no association, while others found a positive or negative association. In addition, juveniles with high scores on all three dimensions of the YPI-S (psychopathic-like juveniles) showed more conduct problems and offenses than their non-psychopathic-like counterparts. In line with the original 50-item YPI, these results were found in both the general population and offender samples of both genders [1, 16, 18, 27, 30, 47–50, 58, 62].

Finally, two studies have not been limited to adolescence, but have also tested the use of the YPI-S in emerging adulthood. Hawes and colleagues [34] examined the stability of psychopathic traits as measured with the YPI-S from adolescence to young adulthood (from 17 to 24 years). They found that these traits decreased over time, but remained invariant. The relationships with relevant functional outcomes, such as personality, mental disorders and offending behavior were temporarily consistent and in line with what could be expected based on theory. Colins and Andershed [14] tested the psychometric properties of the YPI-S in a Swedish sample of young adults (aged 20–24 years). In line with reseach in adolescent samples, they found the aformentioned three-factor strucutre, acceptable internal consistency (although the internal consistency for the affective dimesion in females and the behaviorial dimension in males as well as in the total sample were only marginal), and correlations with externalizing MHP, substance use problems and offending behavior.

### Current study

Recently, our research group constructed the German version of the YPI-S based on the German translation of the original 50-item YPI [3, 5, 37, 54, 57]. However, this German version of the YPI-S has not been validated yet. The aim of the current paper is, therefore, to examine the psychometric properties of the German version of the YPI-S in the German-speaking part of Switzerland. In line with previous research, we expect to replicate the three-factor structure consisting of the GM, CU, and II dimensions, moderate to good internal consistency of the total score and the three factors, MI across gender, a positive relationship with externalizing MHP, substance abuse problems, and offending behavior. As previous results regarding the association between the YPI-S and internalizing MHP are mixed, we presume no relationship, although a small positive or negative relationship is not inconceivable.

## Methods

### Procedure

For the current study, the sample was recruited from 18 public schools covering all curricula in both urban and rural areas from the German-speaking part of Switzerland. Principals of these schools were contacted by a member of the research team and asked for the opportunity to present the study to their students. All contacted principals agreed. A few days prior to assessment, a research assistant attended a one-hour class to present the study to the students and handed out written information on the study. Adolescents between 15 and 19 years with sufficient knowledge of the German language to complete the questionnaires were eligible for inclusion. Written informed consent (including a written informed consent of their parents or legal guardians when underage) was obtained. Students completed self-report questionnaires regarding psychopathic traits, MHP and offending behavior. These were obtained to compare previously acquired data in high risk samples (e.g., juveniles in residential care) with data in the general population. The assessments took place during a one-hour class. Participants had the chance to win movie tickets. The study was approved by the Ethics Committee of the Cantons of Basel-Stadt and Basel-Landschaft.

### Participants

For the current paper, data from 856 adolescents aged 15–19 years (*M*_age_ = 17.18; *SD* = 1.18) of the German-speaking part of Switzerland were included.^1^ The sample consisted of 57.0% (*n* = 488) boys and 43.0% (*n* = 368) girls. All participants completed the YPI [3], of which we derived the 18 items of the YPI-S in accordance with Van Baardewijk et al. [59]. Furthermore, the Massachusetts Youth Screening Instrument-Version 2 (MAYSI-2; ([31, 32]; German translation: [55]), as well a self-reported delinquency questionnaire adapted from the Münster longitudinal study [7] were also assessed.

### Instruments

#### The Youth Psychopahic Traits Inventory-Short Version (YPI-S)

The YPI-S ([59]; German translation: [60]) is a 18-item self-report questionnaire derived from the 50-item original YPI [3, 5]. In accordance with the 50-item original version, the individual items are categorized into three dimensions: the interpersonal, or grandiose-manipulative, dimension, the affective, or callous-unemotional, dimension, and the behavioral, or impulsive-irresponsible, dimension. In the YPI-S, items are not subdivided into the ten subscales of the YPI. Items are rated on a 4-point Likert scale ranging from 1 = does not apply at all to 4 = applies very well. As mentioned in the introduction, the reliability and validity of the YPS-S are promising and seem to be in line with the original 50-item YPI [1, 14, 16, 18, 27, 30, 47–50, 58, 62].

#### The Massachusetts Youth Screening Instrument-Version 2 (MAYSI-2)

The MAYSI-2 ([31, 32]; German translation: [55]) is a 52-item screening tool to identify youth (aged between 12 and 17 years) who are at immediate risk for suicide, and increased mental health and substance use needs. The instrument was originally developed for the use in the juvenile justice system, but is today also used in other samples (e.g., youth welfare, general population) at least in Switzerland. The MAYSI-2 contains seven scales: *Alcohol/Drug Use (ADU)*, *Angry-Irritable (AI)*, *Anxious-Depressed (AD)*, *Somatic Complaints (SC)*, *Suicide Ideation (SI)*, *Thought Disturbance (TD)*, and *Traumatic Experiences (TE)*. All scales, except for the *TE* scale have a caution cut-off and a warning cut-off. A score above the caution cut-off suggests clinical significance, whereas a score above the warning cut-off indicates higher scores than those rendered for 90% of juveniles in a normative sample. Research has shown adequate reliability and validity [33]. For the current study, we included the ADU (Cronbach’s α = 0.65; MIC = 0.55), AI (Cronbach’s α = 0.84; MIC = 0.40) and AD (Cronbach’s α = 0.84; MIC = 0.38) scales.

Self-reported delinquency questionnaire adapted from the Münster longitudinal study (further the self-reported delinquency questionnaire). The self-reported delinquency questionnaire is an adapted questionnaire based on the delinquency questionnaire used in the Münster longitudinal study [7]. It was used to assess the lifetime prevalence of the adolescents’ delinquent behavior. The questionnaire assesses three categories of delinquent behavior (vandalism (3 items), property offenses (8 items), and violent offenses (4 items)). Participants were asked if they had ever committed a certain type of offense (yes/no). Additionally, we computed a variable indicating delinquent versatility (i.e., number of different offenses; possible range: 0–15).

### Statistical analysis

Analyses were conducted with SPSS 25 (IBM Corp. Released 2017) and Mplus (Version 6.12 [43];). The level of significance was set at .01 to account for the large number of analyses. First, we will present descriptive statistics for the YPI-S, MAYSI-2, and offending behavior for the total sample, as well as for boys and girls separately. Gender differences were examined using *t-* or χ^2^-tests respectively. Furthermore, effect sizes (Cohen’s *d*) were calculated to evaluate the magnitude of gender differences, with values between .20 and .49 indicating a small effect, values between .50 and .79 indicating a moderate effect, and values of and above .80 indicating a large effect [13].

Second, we conducted a confirmatory factor analysis (CFA) with categorical indicators using a robust weighted least squares estimator (weighted least squares means and variance adjusted; WLSMV) for boys and girls separately to test the three factor structure of the YPI-S.^2^ Model fit was assessed using root mean square error of approximation (RMSEA; scores below .05 indicating good fit and scores between .05 and .08 indicating acceptable fit) and comparative fit index (CFI; scores between .90 and .95 indicating good fit and scores of and above .95 indicating excellent fit) [36]. To test whether the model was invariant across gender we conducted a multi-group analysis following the steps outlined in the Mplus user’s guide [42]. We used the following guidelines to examine measurement invariance: ∆CFI ≤ .01 [10, 11], ∆RMSEA ≤ .015 [10], and the Tucker-Lewis Index (TLI) for the constrained model should not be lower than for the unconstrained model [39]. However, we also reported ∆χ^2^ and its associated *p*-value.

Third, we calculated Cronbach’s α, mean corrected item-to-total correlations (MCITC), and mean inter-item correlations (MIC). Cronbach’s α between .60 and .69 are considered marginal, between .70 and .79 acceptable, between .80 and .89 good, and .90 and higher are considered excellent [6]. The MCITC should be higher than .30 [45] and the MIC should be between .15 and .50 [12].

Fourth and finally, we calculated zero-order correlations between the YPI-S, the MAYSI-2 and offending behavior, followed by partial correlations controlling for the YPI-S dimensions, since they were correlated. Fisher’s Z was used to test if correlation coefficients differed between boys and girls. To interpret the magnitude of the correlation coefficients, we followed Cohen’s [13] benchmark of a small (r = .10), medium (r = .30) and large (r = .50) effect.

## Results

In Table 1, mean scores of the YPI-S, the MAYSI-2, and lifetime prevalence rates of self-reported delinquent behavior are presented for the total sample, as well as for boys and girls separately. Two third of the total sample reported at least one offense, and one in four students at least one violent offense.

**Table 1:** Descriptive statistics for the total sample and differences between boys and girls

	Total sample (*n* = 856)	Boys (*n* = 488)	Girls (*n* = 368)		
	*M* (*SD*)			*t*; *p*	Cohen’s *d*
YPI-S GM	11.08 (3.68)	11.90 (3.78)	9.99 (3.24)	7.91;< .001	0.54
YPI-S CU	10.58 (3.44)	11.51 (3.45)	9.34 (3.00)	9.76;< .001	0.67
YPI-S II	13.11 (3.09)	13.15 (3.05)	13.06 (3.15)	0.41; .684	0.03
YPI-S Total	34.73 (7.85)	36.51 (8.02)	32.36 (6.95)	8.06; < .001	0.55
MAYSI-2 ADU	1.77 (2.17)	2.22 (2.31)	1.17 (1.79)	7.49; < .001	0.51
MAYSI-2 AI	2.46 (2.23)	2.22 (2.12)	2.79 (2.34)	−3.66; < .001	−0.26
MAYSI-2 AD	1.40 (1.62)	1.07 (1.34)	1.85 (1.84)	−6.86; < .001	−0.49
Delinquent versatility	1.91 (2.32)	2.56 (2.62)	1.04 (1.44)	10.09; <.001	0.69
	% (*n*)			χ^2^; *p*	*OR*
Any offense	66.6 (570)	76.8 (375)	53.0 (195)	53.66; < .001	0.34
Vandalism	30.5 (261)	41.2 (201)	16.3 (60)	61.30; < .001	0.28
Property offense	54.9 (470)	61.3 (299)	46.5 (171)	18.57; < .001	0.55
Violent offense	26.4 (226)	36.7 (179)	12.8 (47)	61.72; < .001	0.25

Notes. *YPI-S* Youth Psychopathic Traits Inventory-Short Version, *GM* Grandiose-Manipulative, *CU* Callous-Unemotional, *II* Impulsive-Irresponsible, *MAYSI-2* Massachusetts Youth Screening Instrument-Version 2, *ADU* Alcohol/Drug Use, *AI* Angry-Irritable, *AD* Anxious-Depressed

Boys reported more GM and CU traits than girls. Boys also had more alcohol and drug use problems, but fewer angry-irritable and anxious-depressed problems than girls. Finally, the prevalence of self-reported offending behavior was higher in boys than in girls.

Results of the CFA indicated an acceptable fit for both boys and girls. The RMSEA and CFI were 0.07 (90% CI: 0.06–0.08) and 0.92 for boys (χ^2^[*df =* 132, *n* = 488] = 437.28, *p* < 0.001; χ^2^/*df* = 3.31), and 0.06 (90% CI: 0.06–0.07) and 0.90 for girls (χ^2^[*df* = 132*, n* = 368] = 331.19, p < 0.001; χ^2^/*df* = 2.51). The standardized and unstandardized factor loadings of the items for boys and girls can be found in Table 2.

**Table 2:** Standardized and unstandardized factor loadings for boys and girls

	Boys			Girls		
Item	β	*B*	*SE*	β	*B*	*SE*
Grandiose-Manipulative						
4	0.77	1.00		0.77	1.00	
5	0.67	0.76	0.08	0.65	0.71	0.12
8	0.68	0.78	0.09	0.51	0.49	0.09
9	0.77	1.00	0.10	0.80	1.12	0.19
14	0.75	0.96	0.11	0.68	0.77	0.12
16	0.60	0.62	0.07	0.49	0.46	0.09
Callous-Unemotional						
3	0.74	1.00		0.68	1.00	
6	0.40	0.40	0.08	0.55	0.70	0.16
10	0.68	0.86	0.13	0.66	0.95	0.17
15	0.52	0.56	0.09	0.45	0.54	0.13
17	0.72	0.96	0.15	0.73	1.15	0.23
18	0.61	0.71	0.11	0.63	0.88	0.16
Impulsive-Irresponsible						
1	0.74	1.00		0.76	1.00	
2	0.46	0.48	0.09	0.47	0.46	0.10
7	0.53	0.56	0.10	0.62	0.68	0.12
11	0.53	0.57	0.10	0.48	0.47	0.09
12	0.69	0.86	0.13	0.64	0.72	0.13
13	0.58	0.65	0.10	0.62	0.67	0.13

Notes. All standardized factor loadings were statistically significant (*p* < .001)

Next, MI across gender was tested (Table 3). The configural model (no constraints included) had an acceptable fit (χ^2^[*df* = 264*, n* = 856] = 757.72, *p* < 0.001; χ^2^/*df* = 2.87). Metric invariance (factor loadings are constrained to be equal across gender) and scalar invariance (loadings and thresholds constrained to be equal across gender) can be assumed based on the CFI, TLI, and RMSEA, although the ∆χ^2^ is significant. However, modification indices for the scalar invariance model indicated that the model fit could further be improved if the thresholds for item 1 (*I have probably skipped school or work more than other people.*) and item 8 (*I have talents that go far beyond other people’s.*) were allowed to vary across gender. Strict invariance (item residual variances constrained to be equal across gender) can also be assumed based on the CFI, TLI, and RMSEA.

**Table 3:** Summary of goodness-of-fit statistics for gender invariance models

Model	∆χ^2^ (df) ^a^	CFI	TLI	RMSEA
Configural invariance		0.915	0.902	0.066
Metric invariance	46.42 (15)*	0.911	0.903	0.066
Scalar invariance	100.80 (33)*	0.905	0.907	0.064
Strict invariance	91.61 (18)*	0.896	0.904	0.065

Notes. ^a^ Test for difference testing using the DIFFTEST option in MPlus; *CFI* Comparative Fit Index, *TLI* Tucker-Lewis Index, *RMSEA* Root-Mean-Square Error of Approximation; * *p* < .001

In general, Cronbach’s αs for the total score and the GM dimension were good, acceptable for the CU dimension, and marginal for the II dimension (Table 4). All mean corrected item-to-total correlation (MCITC) values were above the recommended cut off of .30, indicating that excluding items for the total score or one of the three dimensions did not improve the α. Finally, all mean inter-item correlation (MIC) values were between .18 and .42, which is within the recommended range of .15 and .50.

**Table 4:** Internal consistency of the YPI-S scores in the total sample and in boys and girls

	Total sample (*n* = 856)			Boys (*n* = 488)			Girls (*n* = 368)		
	α	MCITC	MIC	α	MCITC	MIC	α	MCITC	MIC
GM	.80	.55	.40	.81	.57	.42	.74	.48	.32
CU	.73	.46	.31	.71	.45	.29	.68	.41	.26
II	.67	.40	.26	.67	.40	.26	.68	.41	.26
Total	.83	.42	.21	.83	.43	.22	.79	.37	.18

*Notes*. *GM* Grandiose-Manipulative, *CU* Callous-Unemotional, *II* Impulsive-Irresponsible, *MCITC* Mean corrected item-to-total correlation, *MIC* Mean inter-item correlation

At the zero-order level, we generally found small to medium positive correlations among the YPI-S and MHP in the total sample, as well as in boys and girls separately (Table 5). We found no relationship between CU traits and alcohol and drug use problems in boys and girls, and between GM and CU traits, and anxious-depressed problems in the total sample and in boys. At the partial correlation level, GM and II traits remained significantly related to alcohol and drug use problems, and II traits remained correlated with angry-irritability and anxious-depressed problems. Remarkably, in girls, GM traits additionally remained associated with both angry-irritability and anxious-depressed problems, and CU traits with angry-irritability problems. Fisher *z* scores showed gender differences for the YPI-S total score and GM traits, and angry-irritability and anxious-depressed problems, with girls showing stronger correlations than boys. In addition, the relationship between CU traits and angry-irritability problems at the zero-order level was also stronger in girls.

**Table 5:** Bivariate and partial correlations between YPI-S scores and MAYSI-2 subscales in the total sample and differences between boys and girls

		Total sample (*n* = 856)				*Boys (n* = 488)				Girls (*n* = 368)				*z*-score			
	Type of correlation	Total	GM	CU	II	Total	GM	CU	II	Total	GM	CU	II	Total	GM	CU	II
MAYSI-2 ADU	Zero-order	.31**	.29**	.15**	.29**	.24**	.22**	.08	.27**	.31**	.28**	.06	.33**	−1.09	−0.92	0.29	−0.95
	Partial		.17**	.01	.19**		.11*	−.04	.20**		.17**	−.06	.25**		−0.88	0.15	−0.76
MAYSI-2 AI	Zero-order	.34**	.24**	.18**	.40**	.31**	.21**	.17**	.36**	.51**	.38**	.31**	.46**	−3.50***	−2.70**	−2.15*	−1.74
	Partial		.05	.05	.33**		.02	.06	.29**		.19**	.18**	.32**		−2.49**	−1.76	−0.48
MAYSI-2 AD	Zero-order	.15**	.09**	.01	.26**	.13**	.07	.03	.22**	.35**	.28**	.16**	.32**	−3.39***	−3.14**	−1.90	−1.56
	Partial		.00	−.06	.24**		−.04	−.02	.22**		.16**	.06	.21**		−2.91**	−1.16	0.15

Notes. *GM* Grandiose-Manipulative, *CU* Callous-Unemotional, *II* Impulsive-Irresponsible, *ADU* Alcohol/Drug Use, *AI* Angry-Irritable, *AD* Anxious-Depressed; ** < .01; *** < .001

In Table 6, the relation between the YPI-S and self-reported delinquency is presented. At the zero-order level, generally small to medium associations were generally found. Non-significant results were found for CU traits and all offense variables in girls, as well as for CU traits and property offenses in boys. At the partial correlation level, in the total sample as well as in boys, GM and II generally remained related. However, regarding violent offending, although CU traits remained correlated, GM and II traits did not. In girls, overall, only II traits remained related with offending behavior. Fisher *z* scores found stronger correlations for II traits and delinquent versatility at the partial correlation level in girls than in boys. In addition, the relationship between GM and versatility at the zero-order level in boys was stronger compared to girls.

**Table 6:** Bivariate and partial correlations between YPI-S scores and self-reported delinquency in the total sample and differences between boys and girls

		Total sample (*n* = 856)				*Boys (n* = 488)				Girls (*n* = 368)				*z*-score			
	Type of correlation	Total	GM	CU	II	Total	GM	CU	II	Total	GM	CU	II	Total	GM	CU	II
Any offense	Zero-order	.31*	.26*	.18*	.29*	.26*	.21*	.17*	.24*	.28*	.22*	.04	.36*	−0**.**31	−0.15	1.90	−1.91
	Partial		.13*	.06	.19*		.08	.08	.15*		.10	−.08	.30*		−0.29	2.31	−2.29
Delinquent versatility	Zero-order	.43*	.40*	.27*	.31*	.40*	.40*	.22*	.31*	.34*	.24*	.11	.39*	1.0	2.58*	1.63	−1.32
	Partial		.26*	.11*	.15*		.26*	.04	.15*		.10	−.02	.33*		2.39	0.87	−2.77*
Vandalism	Zero-order	.29*	.27*	.18*	.22*	.24*	.23*	.13*	.19*	.23*	.18*	.06	.29*	0.15	0.75	1.02	−1.53
	Partial		.16*	.06	.11*		.14*	.08	.10		.07	−.04	.24*		1.02	1.73	−2.08
Property offense	Zero-order	.27*	.25*	.10*	.28*	.25*	.24*	.11	.24*	.25*	.21*	−.01	.34*	0.0	0.46	1.45	−1.58
	Partial		.15*	−.02	.19*		.13*	−.01	.15*		.10	−.13	.29*		0.44	1.74	−2.13
Violent offense	Zero-order	.30*	.25*	.25*	.18*	.26*	.23*	.21*	.17*	.21*	.12	.12	.23*	0.76	1.64	1.34	−0.90
	Partial		.12*	.16*	.07		.12	.12*	.05		.00	.07	.19*		1.74	0.73	−2.05

Notes. *GM* Grandiose-Manipulative, *CU* Callous-Unemotional, *II* Impulsive-Irresponsible; * < .01

## Discussion

The aim of the current paper was to examine the psychometric properties of the German version of the YPI-S, in order to establish the reliability and validity of the instrument. In line with our expectations, we were able to replicate the three-factor structure and found marginal to good internal consistency for the total score and the three dimensions. Furthermore, we found positive small to medium correlations with both internalizing and externalizing MHP, substance abuse problems, and offending behavior. Hence, it can be concluded that the German version of the YPI-S seems to be a reliable and valid screening instrument for psychopathic traits for both boys and girls in the general population in the German-speaking part of Switzerland.

Our results are generally in line with the results of previous YPI-S studies: other researchers also found higher psychopathic trait scores in boys than in girls, except for the II dimension [18, 27, 49]. Given the MI in the current study, the results of boys and girls may be compared and girls actually seem to have lower scores on psychopathic traits (except for II) as measured with the YPI-S than boys. Furthermore, the previously described three-factor structure ([1, 16, 18, 27, 30, 47, 48]; Pechorro, da Silva, et al., 2017; Pechorro, Gonçalves, et al., 2017 [59];), and generally marginal to good internal consistency [16, 18, 27, 47–50, 59] were found. Results regarding the concurrent validity were less consistent. Although our results generally pointed to the same direction as Vahl et al. [58], who also used the MAYSI-2, we were not able to find a correlation between CU traits and alcohol and drug use problems, and GM and CU traits and angry-irritable problems. These differences could possibly be explained by the difference in study sample, i.e. general population versus detainees. Furthermore, regarding the relationship between the YPI-S and external MHP as measured with instruments other than the MAYSI-2, we also generally found similar results [16, 18, 30, 48, 50, 58], although the role of II compared to GM and CU was more pronounced in our study (similar to [18]). In line with other studies [30, 48–50], the YPI-S (especially the GM and II dimension) was related to alcohol and drug use problems. Concerning internalizing MHP, some studies were consistent with our results [58], whereas other studies found no correlation [48] or mixed results (often a positive relationship between II and internalizing MHP and a negative relationship between GM and CU and internalizing MHP [16, 18, 30, 50]. Finally, concerning antisocial/delinquent behavior, our results generally corresponded with the results from previous studies [16, 30].

In addition to comparing the German version of YPI-S with the YPI-S in other languages, it is also important to compare the German version of YPI-S with the German version of YPI. Compared to the 50-item YPI, the YPI-S had a weaker internal consistency. In addition, unlike in the YPI-S, boys and girls did not only differ on the GM and CU dimension, but also on the II dimension. Finally, the relationship between the YPI and self-reported delinquency was even more evident than in the YPI-S [57].

Furthermore, psychopathic traits (i.e. total score and dimension scores) seem to have a stronger link with internalizing problems in girls than in boys. Our results showed significantly stronger relationships between the YPI-S total score and GM traits, and anxious-depressed problems in girls than in boys. These results are in contrast with previous results regarding the YPI-S in general population samples with direct comparison between boys and girls (i.e., no gender differences between psychopathic traits and emotional problems [18] and a negative relationship between GM traits and social anxiety in girls, but not in boys [49]. With regard to offending samples using the YPI-S and looking into its relationship with internalizing MHP, only the study of Gillen et al. [30] included both boys and girls. However, they only presented results for the total group and did not differentiate by gender. As results are mixed, we feel that more attention should be paid to the relationship between psychopathic traits and internalizing mental problems, especially with the information regarding the higher levels of victimization in female juvenile offenders compared to male juvenile offenders from the previous paragraph [46] in mind. Therefore, it is important not to overlook internalizing MHP when assessing psychopathic traits, especially in girls.

### Limitations

The results of the current psychometric paper should be seen in the light of some limitations. First, our results are based on a study using the 50-item YPI. For the current paper, we extracted the 18 items of the YPI-S and conducted the analyses. However, our results might be influenced because of the relative lengthiness of the original YPI. Therefore, results should be replicated in another study using the YPI-S, instead of extracting the 18 items from the original YPI. Second, our results are based on self-report measures (YPI-S, MAYSI-2, self-reported delinquency questionnaire). This may have biased our results, for example, socially desirable or manipulative answers on the YPI, underreporting of externalizing MHP on the MAYSI-2, overreporting of status-enhancing offenses (property offenses) and underreporting of status-decreasing offenses or shared method variance, which could inflate relationships. Finally, unfortunately we were not able to validated the YPI-S with a golden standard instrument for psychopathy, such as the PCL:YV [26]. Future validation studies with the YPI-S in German-speaking countries could take this into account.

### Implications

Our results suggest that the German version of the YPI-S seems to be a reliable and valid screening instrument for psychopathic traits in both boys and girls from the general population in the German-speaking part of Switzerland. However, when assessing psychopathic traits, it is important to also pay attention to internalizing MHP, especially in girls. Finally, future studies need to replicate our results using the YPI-S, instead of deriving the items from the original 50 item version, and in other German-speaking countries (i.e., Germany and Austria). In addition, more research regarding gender differences is warranted.

## Conclusions

Our results suggest that the German version of the YPI-S is a reliable and valid screening instrument for psychopathic traits in both boys and girls from the general population in the German-speaking part of Switzerland.

## Data Availability

The data that support the findings of this study are available from the corresponding author upon request.

## Abbreviations

ADU: Alcohol/Drug Use
AI: Angry-Irritable
AD: Anxious-Depressed
CU: Callous-Unemotional
CFI: Comparative Fit Index
CFA: Confirmatory Factor Analysis
GM: Grandiose-Manipulative
II: Impulsive-Irresponsible
MAYSI-2: Massachusetts Youth Screening Instrument-Version-2
MCITC: Mean Corrected Item-to-Total Correlations
MIC: Mean Inter-item Correlations
MHP: Mental Health Problems
RMSEA: Root Mean Square Error of Approximation
SC: Somatic Complaints
SI: Suicide Ideation
TD: Thought Disturbance
TE: Traumatic Experiences
TLI: Tucker-Lewis Index
YPI: Youth Psychopathic traits Inventory
YPI-S: Youth Psychopathic traits Inventory-Short Version

## Footnotes

1 Of the 940 adolescents who were eligible to be included in the study, 84 were excluded for various reasons: refusal study participation (*N* = 36), no informed consent parent or legal guardian (*N* = 11), non-completion of the YPI (*N* = 2), and age outside the age range of the study (*N* = 35).

2 Although students were nested within schools, we did not assume that attending the same school would have a substantial influence on students’ self-report of psychopathic traits. Multilevel analysis should be considered when intraclass correlations (ICC) are larger than .05 [53]. The overall ICC in this study was 0.02.
